# The London Practice of Midwifery; Including the Most Important Diseases of Women and Children. Chiefly Designed for the Use of Students and Early Practitioners. The Sixth Edition, with Alterations, Additional Practical Remarks, and New Sections

**Published:** 1834-01-01

**Authors:** 


*The London Practice of Midwifery; including the most important Diseases of Women and Children. Chiefly designed for the Use of Students and Early Practitioners. The Sixth Edition, with Alterations, additional Practical Remarks, and new Sections.* By Geo. Jewel, m.d. &c.—London, 1833. 12mo. pp. 407.

This work has been so long known to the profession, and has attained so great a degree of popularity, that it is scarcely necessary for us to do more than announce to our readers that another edition has just been published, under the superintendence of Dr. Jewel. New sections of considerable length, on the subjects of Leucorrhœa, Spontaneous Evolution, Puerperal Mania, and Puerperal Fever, have been introduced, and many short but useful practical remarks have been inserted throughout the volume.

Publications on midwifery have wonderfully increased of late: we do not, however, object to their multiplication, believing most firmly that great advantage must necessarily be derived from them; for, while the general outline is pretty much the same, there is an endless variety in the details; one author selecting particular subjects upon which he dwells at length, while the attention of another will be directed to different though equally important points. A man, therefore, who wishes for complete information on any given subject ought to peruse almost every work connected with it, and not to suppose that, because their titles are the same, the contained matter must necessarily be the same also.

It is well known that Dr. Jewel has paid considerable attention to leucorrhœa, and has published a very practical essay on the subject. So common is this discharge, that it may be said with truth that few women are free from it: it arises from very different, and even opposite causes, and therefore requires great discrimination in its treatment. We perfectly agree with the author, that the *seat* of the disease is various: he states,

"The discharge most commonly arises from the upper part of the vagina and cervix uteri, but in some cases it may be traced to a high degree of irritation, if not inflammation of the subacute kind in the cervix, a state of the complaint which is often mistaken for carcinoma uteri in its incipient stage, and consequently no remedies are prescribed, or a very inefficient mode of practice is adopted; and, although the local symptoms may not be dissimilar, yet, in this complaint, the os uteri will not be found opened to the same extent as in carcinoma, nor will its margin present the same cartilaginous hardness to the touch. It should be borne in mind that profuse leucorrhœa occurring at the period of life when the uterus is about to cease in the performance of its natural function, is oftentimes indicative of structural disease, and hence the necessity of a careful examination. The predisposing and exciting causes of this complaint are various. Among the most prominent may be mentioned a scrophulous diathesis, frequent child-bearings or abortions, a disordered state of the menstrual function, &c. Ascarides in the lower part of the intestinal tube will give rise to a vaginal discharge. It has also been thought to arise from the state of the spinal cord, which furnishes the nerves of the uterus." (P. 45.)

Where the discharge proceeds from the mucous follicles around the os uteri, it presents a peculiar dense and creamy appearance, by which it is easily distinguished, and will then commonly be found to depend upon inflammation, either acute or chronic, of those glands, and will consequently require for its cure a certain extent of antiphlogistic remedies. We believe that Sir Charles Clarke was the first to describe this characteristic symptom. The common forms of leucorrhœa are, however, vaginal affections, consisting of an increase in quantity, and an alteration in the quality, of the natural secretion; and, although unquestionably they are frequently the result of debility in the secerning vessels, and hence in these cases the term "weakness" may be justifiably employed, yet they will sometimes be the result of inflammatory action; and on the existence of either of these states depends the proper method of treatment. Dr. Jewel's directions are the following:

"In the treatment of leucorrhœa, attention should be paid to the state of the circulation and general health; for, upon the existing condition of the system must depend very materially the adoption of means likely to effect a removal of the local disease. If a female labours under a vaginal discharge with a full, quick pulse, a coated tongue, thirst, and probably a determination to the cerebral vessels, the abstraction of blood from the arm, together with active purgation and a vegetable diet, would, in all probability, remove the complaint, without the employment of local remedies. It is not often necessary, under the ordinary circumstances of vaginal discharge, to take blood from the arm. Even when apparently admissible, local bleeding, either by the application of leeches or cupping-glasses to the loins, is to be preferred. From the sympathy which exists between the uterine system and the stomach, the functions of this organ soon become imperfectly performed. There will be a sinking at the pit of the stomach, with occasional fulness and palpitation. The general treatment of the patient is now carefully to be attended to, and a rigid system of dietetics will be necessary. Almost the first important step in the cure of leucorrhœa, is a free evacuation of the alimentary canal; and in the use of aperients, we should be governed by the peculiar features of the case. If the digestive functions are impaired, medicines of the purgative kind should be mild in their operation. With regard to local applications, it must be observed, that the various astringents usually employed are very uncertain in their effects. Those commonly in use are a solution of alum, of the sulphate of zinc, of copper, the decoction of oak bark, &c. The editor has introduced into practice a local remedy, which he feels confident will prove more efficient than any other hitherto employed, provided it be judiciously used, namely, the nitrate of silver; and he conceives it has this great advantage, namely, in producing a new action in the part from which the secretion has its origin. It may be applied direct to the part affected by means of the speculum or dilator, or used in the form of injection, commencing with the solution in the proportion of about three grains to the ounce of distilled water, gradually increasing the strength. To prevent the decomposition which takes place when the pewter syringe is employed, the curved bone syringe may be used, which the editor has invented. The patient should place herself in the recumbent posture, and should remain in that position several minutes after the syringe has been withdrawn. The nitrate of silver gives neither pain nor irritation, at least no more than is occasionally produced by the injection of any common astringent." (P. 46.)

An increased flow from the vagina is sometimes produced simply by constipation, and is at once cured by any common aperient, without the employment of local means. The secale cornutum has of late been given, and in many cases with apparent success: our own experience, however, is insufficient to enable us to give any decided opinion regarding its merits. In cases unconnected with an inflammatory condition of parts, and not depending upon organic change or malposition of the uterus, we have usually succeeded with astringent injections, combined with the internal administration of the tinct. ferri muriatis, which we consider a very valuable remedy in these affections. The propriety of bleeding from the arm, under any circumstances, is questionable, but leeches to the pudendum are occasionally useful.

When treating of Puerperal Fever, Dr. Jewel has fallen into the common error of describing several diseases under the same name. We believe that the symptoms of this frightful disease are so distinct from those of peritonitis, that no attentive observer can mistake them: they are more of the genuine "typhus gravior," accompanied by abdominal affection. The cerebral symptoms, however, are at first decidedly predominant, and the important point is to determine, at the commencement of the disease, whether it be malignant puerperal fever or simple acute peritonitis; the latter disease requiring the prompt and repeated use of the lancet; while, in the former, depletion, if used at all, must be employed with the utmost circumspection. This is a question which we cannot afford space to discuss, and therefore must rather abruptly dismiss the subject.

The section on Puerperal Mania contains much useful matter. The symptoms of this distressing malady are thus detailed:

"The first indications of its approach are, an increased irritability of temper, disturbed sleep, and a disposition to talk. One peculiar characteristic of the disease is, that in the course of this unusual volubility, the unfortunate patient, however morally or religiously disposed when in health, uses the most improper, and oftentimes indelicate language. In some cases she is obstinately silent. The countenance becomes pallid and anxious, the eye is restless, the tongue is coated, and the pulse is generally accelerated. The editor has known the disease to be ushered in by a pulse beating 120 in a minute, which continued, without any deviation, for a period of four days before the maniacal symptoms became developed. The bowels are almost invariably constipated. The secretion of milk usually continues uninterrupted. This disease may be excited by a very trifling cause, particularly if the patient has had an attack on a previous ocasion, or if there be a tendency to hereditary insanity. In such cases, every thing which may excite the patient after her labour should be most carefully avoided. The state of the bowels should be attended to, the general comfort of the patient studied, and her mind kept constantly and agreeably amused. Uterine hæmorrhage, or any circumstance tending to depress the bodily and mental powers, seriously predisposes to the disease." (P. 340.)

The editor states that the average mortality in this disease is about one in fifteen, but we think this an over-statement. In our own practice we have never had a fatal case where it has been simple puerperal mania. If the affection be combined with phrenitis, then there will be great danger.

In the treatment of this disease our experience coincides with the editor's, and teaches us that active depletion is rarely necessary, and caution is required in the use of purgatives; gentle laxatives being much more serviceable than those of a more active kind. We well recollect attending a lady whose symptoms were several times aggravated in consequence of our being obliged to have recourse to powerful aperients, when milder ones had failed. Opiates are in general decidedly hurtful, although in some cases, where there is great nervous irritability, they may be employed with advantage. We would caution our readers against applying a blister "over the back of the head:" in this situation it will sometimes aggravate the symptoms; the nape of the neck is the more proper place for it. When the patient begins to recover, the most powerful tonics are indicated.

Dr. Jewel's additions are clear in style, and useful in substance. As to the original work, it needs no eulogium of ours: we will merely observe, that the beginner may learn from it not only the practice of midwifery, but also the valuable lesson, that to be instructive it is not necessary to be dull.

---

*The Edinburgh Medical and Surgical Journal*, No. CXVII. October, 1833.

